# Social Isolation Induces Sex‐Specific Differences in Behavior and Gut Microbiota Composition in Stress‐Sensitive Rats

**DOI:** 10.1002/brb3.70621

**Published:** 2025-06-10

**Authors:** Charlotte Hurst, Gosia Zobel, Wayne Young, Trent Olson, Nabil Parkar, Jeremy Bracegirdle, Rina Hannaford, Rachel C. Anderson, Julie E. Dalziel

**Affiliations:** ^1^ AgResearch Ltd. Palmerston North New Zealand; ^2^ AgResearch Ltd., Ruakura Research Centre Hamilton New Zealand

**Keywords:** anxiety, depression, elevated plus maze, gut–brain axis, memory, open field test

## Abstract

**Background:**

Social isolation (SI) is an established rat model of chronic stress. We applied this to the stress‐sensitive Wistar Kyoto (WKY) strain to explore brain‐to‐gut interactions associated with mood. Whether SI stress‐induced behavioral changes are sex‐specific or if they affect the microbiome in WKY is unknown. We hypothesized individually housed (IH) animals would be more anxious than pair‐housed (PH), with sex differences. Male and female rats were either IH or PH from 70 to 112 days old and behavior was assessed in modified open field (OFTmod), elevated plus maze (EPM), and novel object recognition (NOR) tests. Cecal content DNA was analyzed by shotgun metagenome sequencing.

**Results:**

IH rats, particularly females, spent more time in the center of the OFTmod where the semi‐novel feed was presented compared to PH group rats. There was a tendency for greater distance traveled, or potential hyperactivity, in IH female rats. Males stayed in the EPM closed arms more than females. No treatment difference occurred for recognition memory. SI altered cecal microbiome composition in females where housing was associated with seven differentially abundant taxa and 49 differentially abundant KEGG Level 3 ortholog/gene categories. Several relationships were noted between behavioral traits and relative abundance of microbiome taxa. There was a greater shift in female microbiome composition.

**Conclusions:**

In summary, behavioral responses to the housing treatment were minimal. IH animals, particularly females, spent more time in the center of an OFT that contained food; this may have been an indication of depression, as opposed to anxiety. Housing status had a differential impact on the microbiome for females compared to males. The associations between cecal microbiota and activity in the modified OFT suggest that dietary interventions that influence the relative abundance of *Bifidobacteria, Alistipes*, and Muribaculaceae should be explored.

## Graphical abstract

Individually housed (IH) and pair‐housed (PH), male and female, Wistar Kyoto rats were assessed for differences in behavior and gut microbiome after 5 weeks of differential housing, anticipating the IH animals would display increased anxiogenic behaviors. No difference was found between the IH and PH males; however, a shift in both behavior and microbiome composition was found in the IH females, who displayed fewer anxiogenic behaviors contrary to our predictions. Behaviors correlated with relative abundance of several microbial taxa.

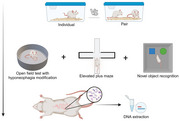

## 1 Introduction

The impact of social isolation (SI) in Wistar Kyoto (WKY) rats remains relatively unexplored, despite being a commonly used model of anxiety, depression (Jiao et al. 2011), and irritable bowel syndrome (IBS) (Dalziel et al. 2017). Importantly, the literature using SI to induce chronic stress is dominated by male rat models, whereas conditions such as anxiety, depression, and IBS disproportionately affect females (McLean et al. 2011). Given this, gaining a better understanding of the impact of SI in females is an important knowledge gap to fill. While a few studies have investigated the microbiome in WKY rats (Bassett et al. 2019; Abboud et al. 2021), the relationship between sex, microbiome, SI, and behavioral outcomes for this model of gut–brain axis dysfunction has not been studied. Through understanding how behavior and the gut microbiota are altered by chronic stress, we can better understand brain‐to‐gut signaling. The aim of this study was to determine how applying a chronic stress paradigm through SI would affect behavior in a stress‐sensitive animal model.

Because rodents are highly social animals, SI during adolescence can cause high levels of chronic stress and alter brain development and behavior in the long term (Hatch et al. 1965; Bianchi et al. 2006; Dunphy‐Doherty et al. 2018; Watson et al. 2012). Such effects have been shown to differ across strain (Hall et al. 2000; Paulus et al. 1998; Fox et al. 2015) and sex (Weiss et al. 2004; Krupina et al. 2020; Lukkes et al. 2009). Other factors that affect behavioral and physiological measures include age (Paulus et al. 1998; Shoji and Mizoguchi 2011), duration of isolation (Ravenelle et al. 2014), housing enrichment (Das et al. 2015), and test conditions (Krupina et al. 2020).

Given the predominance of stress for human females, it is especially important to understand how SI interacts with sex. Typically, the SI model in normo‐sensitive rat strains exposes males to SI for 2–4 weeks and they show increased anxiety, demonstrated by less time spent in the center of the open field test (OFT) and increased locomotor activity (Levine et al. 2007), and similarly in the elevated plus maze (EPM) (Novoa et al. 2021; Chappell et al. 2013). There is evidence for this in females in that SI increases locomotor activity (Sahakian et al. 1977) and anxious behaviors (Chmelova et al. 2019). However, in another study an anxiety effect was found in males (Chappell et al. 2013) but not females (Butler et al. 2014). This effect can vary depending on the test used, with no SI‐induced anxiety found in the OFT for males or females in Sprague‐Dawley (SD) rats, while males exhibited an EPM anxiety response (Weiss et al. 2004).

To model chronic stress in late adolescence/early adulthood, we aged the rats to 60 postnatal days (pnd) then began a full‐time SI protocol, which has been shown to be effective at this age and to result in a hyperactive phenotype in male rats (Begni et al. 2020). Several studies have investigated the gut microbiome in adult male WKY rats (Bassett et al. 2019; Dalziel et al. 2023), yet the relationship between SI, sex, behavioral performance, and microbiome for this important model of brain‐to‐gut axis dysfunction has not been studied. Thus, we examined the effects of SI on late adolescent WKY male and female rats in modified open field test (OFTmod), EPM and novel object recognition (NOR) behavioral tests, as well as fecal corticosterone levels and cecal microbiome (Young et al. 2022), in comparison to pair‐housed (PH) animals. We hypothesized that SI rats would display a greater level of behaviors associated with measures of anxiety, depression, and memory deficit, with differences between sexes.

## 2 Materials and Methods

### 2.1 Animals

Rats (28–35 pnd) were obtained from the Animal Resource Centre (Perth, Australia) and housed in pairs under PC2 quarantine conditions for 30 days, with access to a ring (50 mm book rings, Dixon) (AgResearch Grasslands Ulyatt Reid Facility, Palmerston North, New Zealand). They were provided ad libitum with standard rodent chow diet (Prolab RMH 1800, LabDiet) and water throughout. Housing consisted of high‐top cages (REW, Australia) with a shelter (drop‐in food hopper on its side, Techniplast), and alternating weekly access to either rings or a wheel (30 cm comfort wheel—Kaytee). Rats housed together during quarantine were kept together during the experiment.

### 2.2 Experimental Design

The study design is summarized in Supporting Information 1. Following quarantine, the rats (females: 158 ± 8 g, range: 143–170 g; males: 220 ± 18 g, range: 189–255 g) were allocated to one of four housing treatments: Pair‐housed female (PF) *n* = 16, individually housed female (IF) *n* = 8, pair‐housed male (PM) *n* = 16, and individually housed male (IM) *n* = 8. To facilitate behavior testing, the treatments were split equally into two blocks, the timeline of the second block was offset by 1 week. One day prior to being housed in their trial conditions, all animals underwent an initial (non‐hyponeophagia) OFT (Dalziel et al. 2023) to record their baseline anxiety. The following day they were placed into their housing conditions and were maintained in these conditions for 28 days, followed by behavioral tests over 5 days. Rats were killed by CO_2_ overdose. The cecal contents were collected and snap frozen in liquid nitrogen and stored at −80°C for later analysis.

### 2.3 Behavioral Test Protocol

Behavior testing was carried out during the light phase of the light/dark cycle (between 8:00 a.m. and 12:00 p.m.) in a separate room and video‐recorded using a camera (HERO 7, GOPRO, San Mateo, CA, USA) positioned 250 cm above the test arena. Rats were placed in the test arena each day in the same order and surfaces and objects cleaned with 30% ethanol between animals (Dalziel et al. 2023). Animals were acclimatized in the testing room for 30 min prior. The behavior tests were OFT (10 min; prior to study), OFTmod (10 min) with hyponeophagia component (food), EPM (5 min), and NOR (5 min).

The OFT was conducted in an opaque arena (90 cm diameter, 50 cm high; lux: 700 at arena center). The duration of time that each rat spent in the center of the standard OFT arena (prior to enrolment in housing treatments) was used as an indicator of pretrial baseline anxiety and exploration (McKernan et al. 2010); it was included as a covariate in each behavior model. The OFTmod differed in that it contained home cage food mashed with water (“semi‐novel feed”) in the arena center. This test modification challenged the animal with the opportunity to access familiar food in a novel and confronting environment. The EPM (Panlab, Harvard Apparatus) was performed for 5 min (lux: 200 for open arms, 60–90 for closed arms) to capture behaviors indicative of anxiety. The NOR test was used to test recognition memory and carried out over 3 consecutive days. Lighting for the NOR (opaque rectangular arena 42 cm × 60 cm × 38 cm) was 60 lux in the center of the area. On Day 1, the rats underwent habituation where they were allowed to explore the empty arena for 10 min (Bevins and Besheer 2006). On Day 2 (pretest day), they were introduced to two identical objects (circular opaque grey tube: 15 cm high × 7 cm diameter, 0.5 cm thick, with an open top) for 5 min. On Day 3 (main test day), the object on the left side was switched for a different one (square‐shaped opaque white tube: 15 cm high, 7 ×7 cm^2^, 0.5 cm thick, with an open top) for 5 min. The object was put on the left or right side of the arena for alternating animals.

Videos were analyzed using EthoVision XT 15 (Noldus, Wageningen, The Netherlands, RRID:SCR_000441). Variables for the OFT and EPM test were recorded using the center‐point of the rat as the reference point. For the NOR, the nose‐point was used for the analysis of exploration of the objects. EthoVision automatically recorded the following variables: mean velocity (cm/s), max velocity (cm/s), total distance moved during the test (cm), total frequency of movements during the test (no.), and duration (s) and frequency (no.) spent in each of the zones of interest (OFT: two zones; EPM: open arms, closed arms, center point). For NOR, using the Day 3 results only, the duration of time spent with the novel object versus the familiar object was used to calculate the relative duration. The experimenter conducting the video analysis was blinded to treatment.

### 2.4 Microbiome

Metagenomic DNA was extracted from cecal content using Macherey Nagel NucleoSpin Soil kits (Düren, Germany) and sequenced on an Illumina Novoseq 6000 instrument, generating 150‐bp paired‐end reads, as previously described (Young et al. 2022). Reads were quality trimmed, read pairs joined, and host reads removed as previously described (Dalziel et al. 2023) with the modification of the *Rattus norvegicus* genome (Rnor 6.0 release 102) used as a reference. The Meganizer function from MEGAN6 Ultimate Edition (MEGAN, RRID:SCR_011942) was used for taxonomic binning and to assign putative functions to the DIAMOND alignment files against the KEGG (Kyoto Encyclopedia of Genes and Genomes, RRID:SCR_001120) database (Huson et al. 2016).

### 2.5 Corticosterone

In‐cage fecal sampling was used to avoid blood sampling stress. Two days after each weekly cage change, a 4 mL cryovial (Cryo.sTM) was filled with randomly selected fecal samples from the cage floor. The exception was Week 6, collected 2–4 h after the last behavior test. These were snap frozen in liquid nitrogen and stored at −80°C until analyzed. Feces were freeze‐dried in a Labconco FreeZone 12 Liter Console Freeze Dry System with Stoppering Tray Dryer over a weekend. Dried feces were then ground with a mortar and pestle to a fine powder. Two miligram of powder was reconstituted in 2 mL of absolute EtOH and shaken vigorously for 30 min, centrifuged at 5000 rpm for 15 min, and the supernatant was recovered. Corticosterone was assayed (Invitrogen Corticosterone Competitive ELISA Kit) and absorbance at 450 nm read (Thermo Scientific Multiskan GO Microplate Spectrophotometer).

#### 2.5.1 Statistical Analysis

Data were analyzed using R statistics software (behavior, corticosterone data—v4.3.1; microbiome data—v4.2.0), with data tidying using functions from the R packages tidyverse (Wickham et al. 2019) and janitor (Firke 2023.), and data visualization using the viridis color palette (Garnier et al. 2023.). For behavior tests (Supporting Information 2 for model and assumption details), the statistical significance of the predictors was assessed using likelihood ratio chi‐square tests at the 5% significance level using the analysis of variance function from the lmerTest package (Kuznetsova et al. 2017) for the linear mixed models and in the car package (Fox and Weisberg 2019) for the beta mixed models. Interactions were only included in final models if significant. In the case of evidence of a trend (at the 10% significance level), we used post hoc tests to explore differences between the treatments with the emmeans function from the package with the same name (Lenth 2023.), using Tukey's correction to control for multiple comparisons.

#### 2.5.2 Microbiome

Differences in the abundances of individual taxa and KEGG orthologs/gene functions between pair and individually housed (IH) rats were analyzed using the ANCOM‐BC (Analysis of Compositions of Microbiomes with Bias Correction) package (Lin and Peddada 2020), with each sex analyzed separately. Overall differences in microbiome communities were analyzed by permutation multivariate analysis of variance using the analysis of similarities (ANOSIM) function from the vegan package (Oksanen et al. 2022.). To illustrate taxonomic composition and KEGG abundances within the treatments, principal coordinates analysis plots were calculated using the cmdscale function.

Correlations between behavior variables and the most abundant cecal microbiota genera, (e.g., taxa that made up > 7.5% of the community, providing sufficient coverage and detail), were performed using the corr.test function from the psych package (Revelle 2023) for R. Correlations were visualized using the heatmap.2 function from the gplots (Warnes et al. 2022) package for R. *p < 0.05 represents significant correlations.

#### 2.5.3 Corticosterone

The cage‐level effect of sex and housing (and their interaction) on fecal corticosterone (pg/mL) changes between Week 1 and Week 3 was assessed using a linear model, with block as a covariate. Due to acute stress (from behavior testing), changes between Week 6 were not modeled to assess chronic stress, but raw data were plotted. The difference between Weeks 3 and 6 were measured to detect any remaining acute stress response.

## 3 Results

### 3.1 OFT

*Baseline anxiety*: Pre‐treatment, all animals displayed a strong tendency toward the edge of the arena, potentially precluding discrimination among housing treatment associated anxiety levels (Supporting Information 3). This was attributed to the high stress sensitivity of the WKY strain. We therefore employed the OFTmod method for the posttreatment OFT test, adapted from a previously reported method (Hermes et al. 2011). Time spent in the center of the OFT and distance traveled during the pretreatment did not differ with either housing or sex (Supporting Information 3). Time spent in the center from the pretreatment OFT was used as a covariate in each behavior model (Supporting Information 2). We note that while this was informative for the OFT, this did not make a difference for the EPM dataset.

**FIGURE 1:**
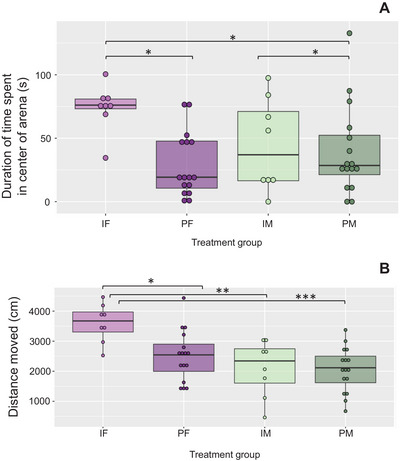
Duration of time spent in the center (A) and total distance moved (B) in the modified open field test (OFTmod) arena for individually housed females (IF, *n* = 8), pair‐housed females (PF, *n* = 16), individually housed males (IM, *n* = 8), and pair‐housed males (PM, *n* = 16). The OFT test was modified by placing a semi‐novel food (home cage feed, mashed in water; new texture) in the center of the novel arena. Tests were 10 min in duration. Significant differences are indicated by *(*p* < 0.05), **(*p* < 0.01), and ***(*p* < 0.001).

*Time spent in center*: Following 4 weeks of housing treatment, IH group rats spent more time in the center of the OFTmod (where the semi‐novel feed was presented) compared to PH group rats (60 ± 7.8 s vs. 34 ± 6.4 s, *p* = 0.014), an effect particularly noticeable in the female animals (Figure 1a). Animals with lower baseline anxiety tended to spend more time in the center of the OFTmod posttreatment (*p* = 0.09).

*Distance traveled*: There was a trend of IH animals moving greater distances if the animal was female (*p* = 0.093). As seen in Figure 1b, compared to IH females (3612 ± 285 cm) other treatments moved less in the modified OFT (PM: −1647 ± 376 cm, *p *< 0.001; IM: −1495 ± 403 cm, *p* = 0.002; PF: −1071 ± 368 cm; *p* = 0.014).

### 3.2 EPM

**FIGURE 2:**
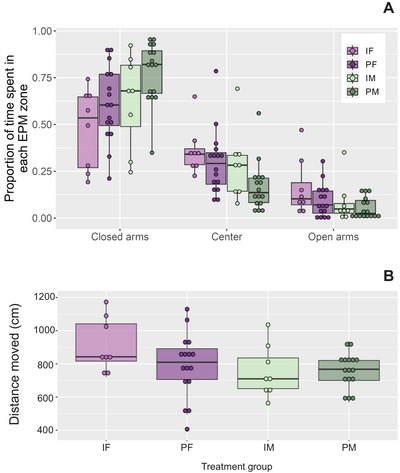
Proportion of time spent in each zone (A) and total distance moved (B) in the elevated plus maze (EPM) for individually housed females (IF, *n* = 8), pair‐housed females (PF, *n* = 16), individually housed males (IM, *n* = 8), and pair‐housed males (PM, *n* = 16). Tests were 5 min in duration.

*Proportion of time per EPM zone*: All four groups of animals spent most of their time in the EPM closed arms, with observed differences due to sex (*p *< 0.001) and housing (*p =* 0.002) (Figure 2a). An animal that was male was more likely to stay in the closed arms compared to their female counterparts (M: 0.71 ± 0.03; F: 0.55 ± 0.04, *p* = 0.014). IH animals tended to spend a lower proportion of their time in the closed arms (0.56 ± 0.05), compared to the PH animals (0.70 ± 0.03*, p* = 0.09).

*Distance moved*: There was no evidence of treatment (*p* = 0.34) or sex (*p* = 0.11) effects for total distance moved in the EPM.

### 3.3 Novel Object Recognition

**FIGURE 3:**
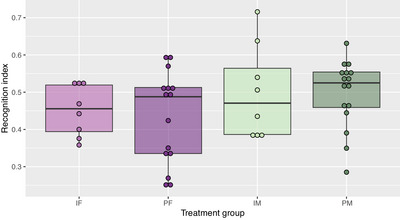
Recognition index (calculated as the time spent with the novel object divided by the total exploration time for both objects) from the novel object recognition (NOR) test for individually housed females (IF, *n* = 8), pair‐housed females (PF, *n* = 16), individually housed males (IM, *n* = 8), and pair‐housed males (PM, *n* = 16). Tests were 5 min in duration.

There was no effect of sidedness (*p* = 0.64) or housing on recognition memory (*p* = 0.52), but there was weak evidence of a sex effect (Figure 3). Male rats (0.50 ± 0.02) tended to have a higher recognition index (F: 0.45 ± 0.02, *p* = 0.093).

### 3.4 Corticosterone Levels

**FIGURE 4:**
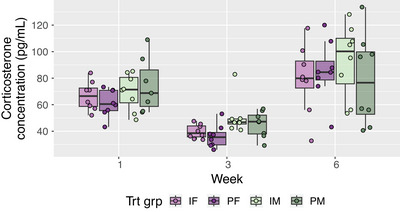
Fecal corticosterone concentration (pg/mL) collected from each cage (*n* = 8 per housing treatment) on Weeks 1, 3, and 6. Week 6 corresponded to the time period where rats were handled for behavior testing. Treatments were individually housed females (IF, *n* = 8), pair‐housed females (PF, *n* = 16), individually housed males (IM, *n* = 8), and pair‐housed males (PM, *n* = 16).

There were no effects of sex (*p* = 0.88) or housing (*p* = 0.43) on cage‐level fecal corticosterone during the study, albeit the data did show elevated corticosterone (< 0.0001) levels across all treatment groups in Week 6 (compared with Week 3), which suggests these measurements were able to capture the impact of any acute stress associated with the behavior tests (Figure 4).

### 3.5 Microbiome Analysis

**FIGURE 5:**
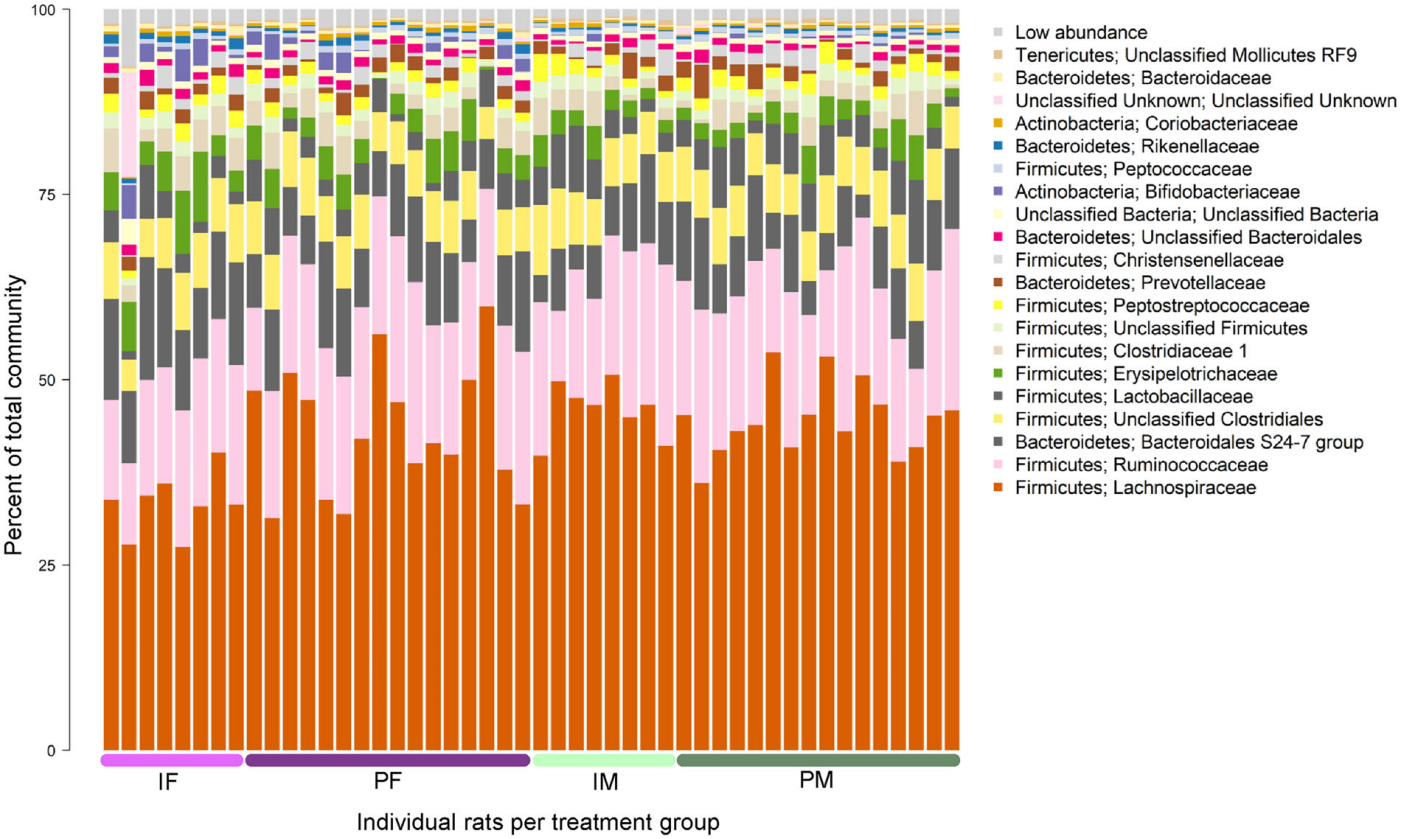
Taxonomic stacked bar plot proportion of the 20 most relatively abundant family level taxa in the cecum of each rat based on treatment (individually housed females: IF, *n* = 8; pair‐housed females: PF, *n* = 16; individually housed males: IM, *n* = 8; pair‐housed males: PM, *n* = 16).

**FIGURE 6:**
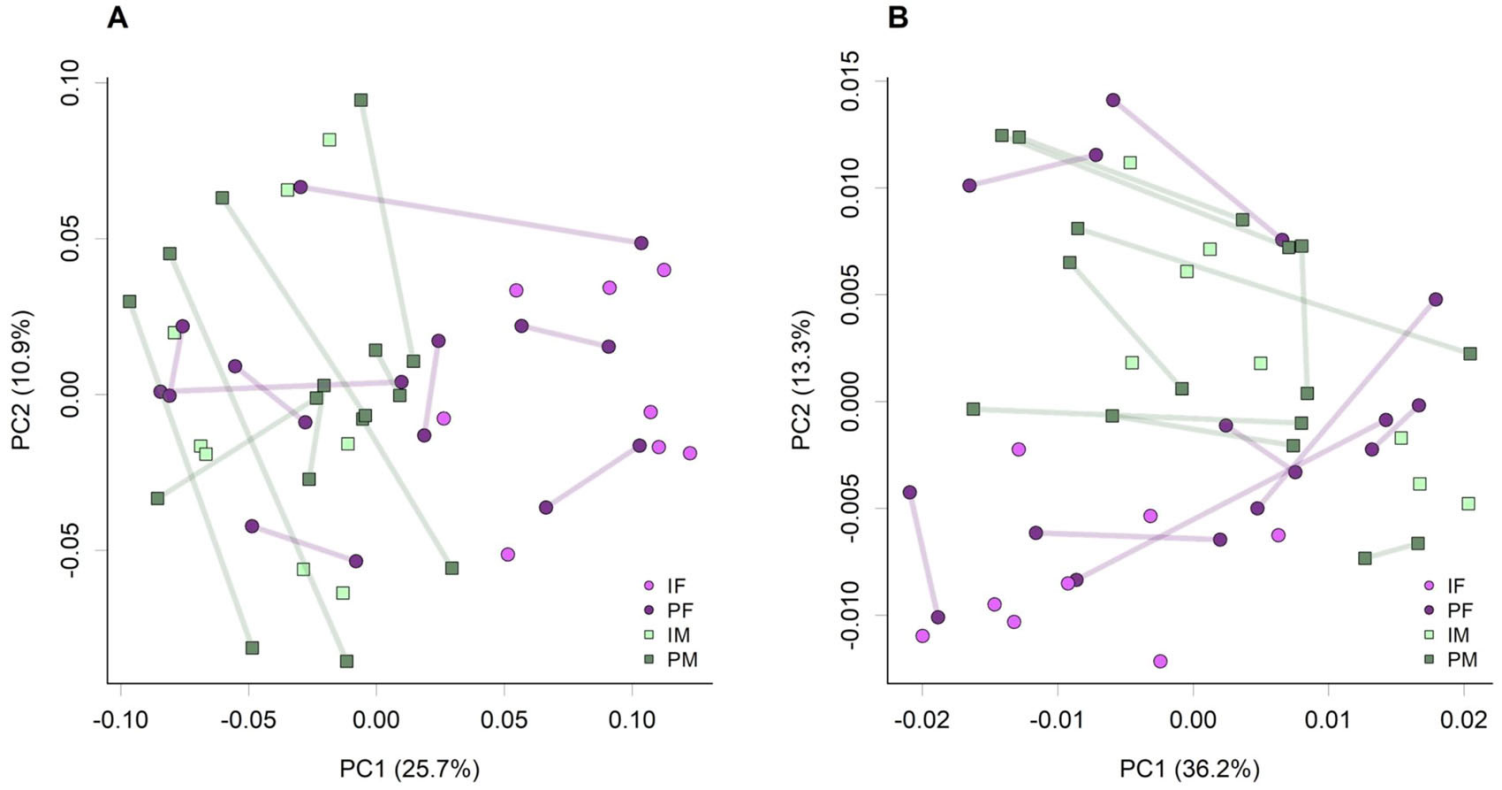
Principle components analysis (PCA) plots of the cecal microbiome taxonomic composition (A) and KEGG Level 4 ortholog (gene function) composition (B) for individually housed females (IF, *n* = 8), pair‐housed females (PF, *n* = 16), individually housed males (IM, *n* = 8), and pair‐housed males (PM, *n* = 16). Shaded lines indicate pair‐mates.

SI altered cecal microbiome composition for both sexes. In male rats, one genus (*Peptococcus*; IM 0.08 ± 0.01% vs. PM 0.28 ± 0.04%, *q *< 0.001) and one KEGG Level 3 gene category, ko00908 Zeatin biosynthesis (IM 0.07 ± 0.001% vs. PM 0.08 ± 0.001%, *q* = 0.048), were differentially abundant. No differences in taxonomic composition were observed between IH and PH for males (data not shown), but comparisons between IH and PH females showed 49 KEGG Level 3 functions were significantly different (Table S1). In female rats (*q *< 0.05), there were seven differentially abundant taxa (Table 1) and 49 differentially abundant KEGG Level 3 gene categories (Table S1). Taxonomic differences included the S24‐7 group (Muribaculaceae family) (IF 12.84 ± 0.875% vs. PF 9.594 ± 0.715%, *q* = 0.001), and *Oscillibacter* (IF 0.65 ± 0.058% vs. PF 1.129 ± 0.144%, *q* = 0.04). Differences in microbiomes for individual rats are presented in the taxonomic bar plot (Figure 5). Taxonomic composition (Figure 6A) and KEGG abundances (Figure 6B) show some separation of the IF rats from the other treatments in terms of microbiome communities. The functional themes were related to microbial transport, microbial signaling and defense, some carbohydrate metabolism genes, metabolism of cofactors and vitamins, and lipid metabolism.

**TABLE 1:** Microbial genera within the cecum with significant differences (ANCOM‐BC, *q* < 0.05) between female rats housed individually (IF, *n* = 8) or in pairs (PF, *n* = 16). Mean percent ± standard error of means.

Phylum	Family	Genus	IF	PF	*p* values	*q* values	W^a^
Firmicutes	Lachnospiraceae	*UCG‐006*	0.11 ± 0.02	0.31 ± 0.07	0.005	0.04	2.808
Firmicutes	Ruminococcaceae	*Oscillibacter*	0.645 ± 0.06	1.13 ± 0.144	0.005	0.04	2.781
Bacteroidetes	Bacteroidaceae	*Bacteroides*	0.59 ± 0.08	0.35 ± 0.05	< 0.001	0.001	−4.033
Bacteroidetes	Muribaculaceae	S24‐7	12.84 ± 0.88	9.594 ± 0.72	< 0.001	0.002	−3.919
Bacteroidetes	Rikenellaceae	*Alistipes*	0.39 ± 0.07	0.26 ± 0.03	0.005	0.04	−2.807
Bacteroidetes	Rikenellaceae	*RC9* gut group	0.47 ± 0.074	0.244 ± 0.06	< 0.001	0.001	−4.27
Bacteroidetes	Unclassified Bacteroidales		1.39 ± 0.17	1.01 ± 0.09	0.004	0.04	−2.884

^a^Test statistic of Analysis of Compositions of Microbiomes with Bias Correction (ANCOM‐BC) package.

### 3.6 Data Integration of Behavior With Microbiota

**FIGURE 7:**
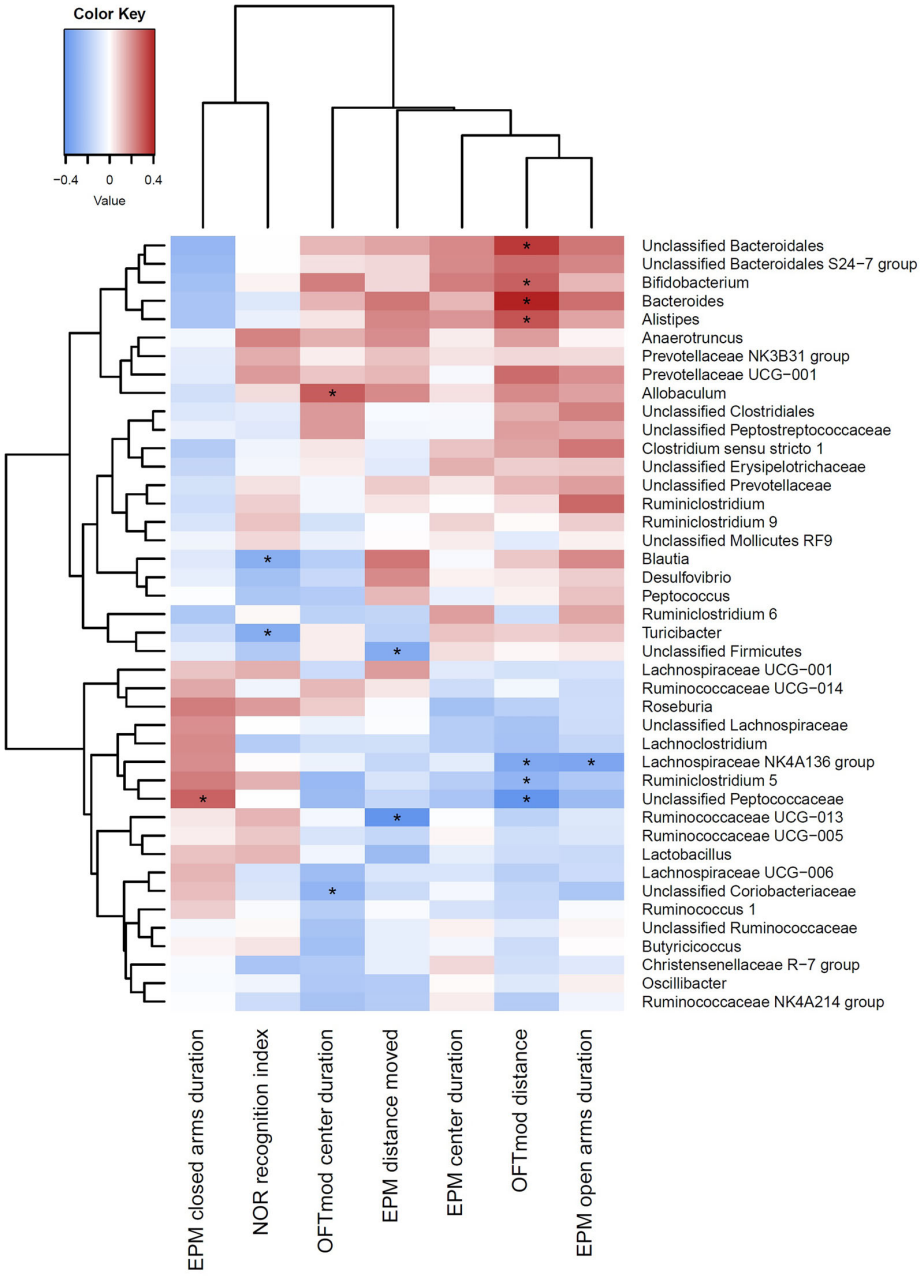
Correlations between behavior variables and the most abundant cecal microbiota genera (defined as taxa that made up > 7.5% of the community). Column ordering is based on similarity of correlations. Treatments were individually housed females (IF, *n* = 8), pair‐housed females (PF, *n* = 16), individually housed males (IM, *n* = 8), and pair‐housed males (PM, *n* = 16). Significant correlations (*P* < 0.05) are indicated by *.

The abundance of several taxa correlated significantly with a range of behavioral variables (Figure 7). These included *Bifidobacterium*, *Alistipes*, and *Bacteroides*, which generally coincided with greater distances moved and/or less time spent in the EPM closed arms. On the other hand, some taxa belonging to subgroups of *Ruminoclostridium*, Lachnospiraceae, Peptococcaceae, and Ruminococcaceae generally coincided with reduced distances and more time spent in the EPM closed arms. Two taxa, *Blautia* and *Turicibacter*, were also found to negatively correlate with NOR recognition index.

## 4 Discussion

The main findings from this study were that SI in the WKY stress‐sensitive rat strain altered behavioral response in a modified (food‐oriented) OFT. There were also shifts in cecal microbial taxa associated with increased abundance of beneficial gut bacteria. Fecal corticosterone levels were anticipated to be lower in PH animals yet were not altered. SI was also expected to decrease recognition memory in the NOR, but we found no difference in the recognition index between groups. Housing largely appeared to have a null effect in behavior tests, apart from IH females spending more time in the center with the food (potentially seeking stimulation) and potentially traveling further (“hyperactivity” frequently attributed to isolation syndrome) in the modified OFT. We acknowledge that any effect of individual housing may have been masked by an effect of separation anxiety experienced by the PH rats when taken for the behavioral testing; separation from a cage mate can cause stress in adult Wistar rats, as measured by a spike in plasma corticosterone levels that occurs regardless of exposure to chronic stress (Ferland and Schrader 2011).

### 4.1 Behavioral Changes Associated With Social Isolation

A higher drive to explore, both open/novel areas and novel foods is suggestive of lower anxiety (Hermes et al. 2011). Contrary to our prediction, in the OFTmod IH female rats spent more time exploring the semi‐novel feed in an open area. This may be because of a difference in the arousal state. Rather than anxious (high arousal state), it is possible that the IH females were depressed (low arousal state) and thus motivated to gain the positive food stimulus, even if this involved going into a potentially aversive location (center of OFT) (Améndola et al. 2022). Individual housing in female hooded rats has been shown to increase locomotor activity when interacting with a novel object (Sahakian et al. 1977). We observed a tendency for further travel in our OFTmod test. The WKY male rats traveled approximately 20 m; this is consistent with that reported previously (e.g., OFT standard—10 min in the same facility; Bassett et al. 2019) (Table S3). Our isolated female rats traveled an even further distance (36 ± 2.8m). Similar distances traveled have been reported in SI and group‐housed male WKY rats (42 vs. 31 m) (Shetty and Sadananda 2017), the former was described as being “hyperactive.” Hyperactivity is often mentioned in relation to “isolation syndrome,” a condition seen when juvenile rats (21–35 pnd; Arakawa 2018) are raised in isolation (Hatch et al. 1965; Dunphy‐Doherty et al. 2018), and has also been reported in late adolescent male Lister Hooded rats (58–101 pnd) (Begni et al. 2020). Hyperactivity is a frequently used, yet unclear term (Dunphy‐Doherty et al. 2018; Yang et al. 2014; S. Li et al. 2020; Syme 1973; Yuan et al. 2019; Botanas et al. 2016; Syme and Hughes 1972). It is important to note that while seeing this effect on activity, consideration is needed for the other anticipated anxiogenic behavioral effects of individual housing. Isolation at 24 days of age resulted in “hyperactive” Lister Hooded rats (Dunphy‐Doherty et al. 2018), yet the authors reported no effect on OFT zone entries, and therefore apparent anxiety levels. Similar results were observed in male WKY rats, isolated at 21 days of age (Shetty and Sadananda 2017). When compared to our PH animals, IH animals spent on average 26 s longer in the center; while a significant difference, in context of the total test (10 min), this is likely to not be as biologically relevant as the dramatically different distances covered in this test between the housing and sex treatments (in particular the isolated females). By measuring the baseline level of anxiety in the OFT, and using this as a covariate, we found that rats that spent more time in the central zone of the arena in the OFT administered prior to housing assignment (e.g., baseline anxiety) tended to do the same in the OFTmod after 28 days of housing treatment, highlighting that underlying individual animal behavioral tendencies may affect the response regardless of treatment.

We anticipated that our results would mirror previous work that reported adolescent male Sprague Dawley SI rats (Weiss et al. 2004) and WKY SI male rats (Shetty and Sadananda 2017) entering EPM open arms less often (Table S2). Instead, we saw a large variation in open arm use across all treatments, suggesting individual animal differences were more important than the treatments. We suggest this may have been due to individual differences in behavior that we did not capture. Améndola et al. (2022) describe the benefits of capturing underlying individual behavior traits and using these as covariates; correspondingly, the behavior we captured in an OFT prior to treatments beginning contributed to explaining some of the variation in OFTmod model. However, it was not useful for the EPM. Thus, we suggest more pre‐study classification of individual differences in behavior for all planned tests may be useful.

We have previously reported a low recognition index in IH adult males (Dalziel et al. 2023), and note that in the current study this was >0.5 for PM. Memory deficits in IH Lister hooded males (25–28 pnd) have been reported (Bianchi et al. 2006) and adult Lister hooded female isolates were able to only recognize a familiar and novel object 1 h after initial exposure, while group‐housed females could discriminate between the objects for up to 4 h (McLean et al. 2010). In male Wistar rats, the retention interval in the NOR was greatly reduced at 48 h compared to 1 h; unfortunately, the result for the 24 h interval was not published (Thanapreedawat et al. 2013). Contrary to our predictions, we found no treatment effect on memory. In fact, most of our rats did not appear to recognize the object; the recognition index was just under or near 50% regardless of treatment, which is similar to other reports (Thanapreedawat et al. 2013); however, social housing of male Sprague Dawley rats (56–70 pnd) has been shown to dramatically increase the recognition index (80%) compared to IH rats (50%), in both a short‐term and long‐term recognition test (Famitafreshi and Karimian 2018). Our test was only conducted at one time point (24 h). We acknowledge that reducing the time between the initial exposure to the familiar objects and the test could have improved the likelihood of identifying an effect of housing conditions on recognition index; however, it is also possible that our results are a reflection of the WKY strain overall memory capabilities.

### 4.2 Corticosterone

No differences in corticosterone were detected from either chronic or acute stress. We did not detect a housing effect in either Week 1 or 3, similar to that reported in a mild stress model in female Sprague Dawley rats (Bear et al. 2023). We scheduled the fecal collection to be 72 h after the weekly cage change, to mitigate its effect on corticosterone levels. However, the 6 week fecal sampling was conducted 2–4 h after the last behavior test. The elevated corticosterone detected (between Weeks 3 and 6 across all groups) was likely that remaining from the acute stress response the previous day, since this peaks at 14–17 h (Bamberg et al. 2001; Lepschy et al. 2007).

### 4.3 Microbiota

The differentially abundant taxa within the phyla Firmicutes and Bacteriodetes in SI females, where Firmicutes decreased and Bacteriodetes increased, demonstrates that housing status influenced how the microbes interact with each other and in turn how they utilize substrates in carbohydrate and lipid metabolism processes. Muribaculaceae have been positively associated with depressive and anxiety‐like behaviors in IH male mice (Liu et al. 2021). Differences in the relative abundance of Rikenellaceae (family) and *Lachnospiraceae bacterium* DW59 (species) have also been reported in female rat stress cohorts, but in the opposite direction (Bear et al. 2023). Similarly, the abundance of *Peptococcus* was lower in SI males, but reported to be increased in male mice exposed to chronic unpredictable social stress (Burokas et al. 2017) and acute stressors, such as restraint and forced swim tests (Gao et al. 2018).

We note that differences exist in microbiota between the WKY depressive phenotype, and normo‐sensitive Sprague Dawley rat strains (IH male rats) in that *Ruminococcus*, *Roseburia*, and unclassified Lachnospiraceae genera are less abundant in WKY rats, whereas the minor taxa *Dorea*, *Turicibacter*, and *Lactobacillus* are higher (Dalziel et al. 2017). Following acute stress from behavioral testing, small changes in the WKY microbiome occur among Firmicutes, particularly *Lactococcus*, with minor increases in *Ruminococcus*, *Roseburia*, and decreased *Lactobacillus* (Bassett et al. 2019).

SI females had increased cecal *Bacteroides* and *Alistipes*, both of which produce gamma‐aminobutyric acid (GABA) (Otaru et al. 2021; Polansky et al. 2016). GABA regulates neuronal excitability in the brain and deficiencies related to depression and anxiety (Prager et al. 2016; Jie et al. 2018). Increased levels of GABA producing bacteria in SI females could have led to resilience (via the gut–brain axis), resulting in fewer anxiety‐related behaviors. *Alistipes* often increased during chronic stress (Lee et al. 2019; Parker et al. 2020; Y. Li et al. 2023), and a higher abundance of this genus can cause dysregulation of the gut–brain axis by decreasing the availability of serotonin, which is associated with depression. Thus, microbiota changes may be indicative of stress at levels of stress insufficient to alter phenotypic behavior.

### 4.4 Correlation Between Behavior and Gut Bacteria

Several taxa's abundances (*Bifidobacterium*, *Bacteroides*, *Alistipes*, and the S24‐7 group, now named Muribaculaceae) were correlated with increased movement in OFTmod (“hyperactivity” frequently attributed to isolation syndrome). However, there was no significant correlation between these taxa and the amount of time spent in the center of the OFTmod (a potential indicator of a low arousal/depression state). Thus, while the study was not designed to capture the nuances between individuals, it is possible that the rats with these gut bacteria avoided depression and instead became hyperactive to cope with the effects of housing and/or the testing regime. Numerous studies have highlighted the potential of *Bifidobacterium* species to reduce stress in animal models and humans (Huang et al. 2022; Y. Li et al. 2022). Different *Bacteroides* species are also able to utilize tryptophan to produce neuroactive metabolites (Zhang et al. 2022) or other metabolites to impact the central nervous system to reduce anxiety‐like behaviors, including capsular carbohydrates such as polysaccharide A (PSA) (Erturk‐Hasdemir et al. 2021) and GABA (Otaru et al. 2021; Polansky et al. 2016).

While neither housing nor sex had any significant effects on the NOR test, significant negative correlations were observed between recognition index and two genera, *Blautia* and *Turicibacter*. The reason these taxa would be less prevalent the higher the recognition index remains unclear, but *Turicibacter* has been shown to promote the release of serotonin (5‐HT) from intestinal cells and can uptake and metabolize 5‐HT (Fung et al. 2019). *Blautia* are commensal bacteria capable of producing SCFA from a variety of sources, including H_2_, carbon monoxide, and carbohydrates (Liu et al. 2021). As such, they are typically thought to play a role in host energy metabolism, including modulating lipid metabolism though conversion of bile acids (Golubeva et al. 2017). However, the role of *Blautia* in the gut–brain axis is less clear, although they have been reported to be elevated in children with attention deficit hyperactivity disorder (Wang et al. 2022), and some species have been shown to promote 5‐HT production and increase motility in the gut (Yano Jessica et al. 2015).

## 5 Conclusions

While a difference between the IH females and the other treatments was demonstrated for time spent in the center, we suggest this may indicate the presence of depression, not anxiety as we had anticipated creating. Isolation did not appear to increase anxiety‐related behaviors in the EPM; however, it is possible that the behavior tests may have created anxiety in the PH rats, masking housing effects. Until this test theory can be tested, we suggest caution in relying on SI to induce anxiety in the WKY rat strain. The associations between cecal microbiota and activity in the modified OFT suggest that dietary interventions that alter the relative abundance of *Bifidobacteria*, *Alistipes*, and Muribaculaceae should be explored.

## Author Contributions

Conceptualization: Charlotte Hurst, Julie E. Dalziel, Gosia Zobel, Rachel C. Anderson, and Wayne Young. Methodology: Charlotte Hurst, Trent Olson, Nabil Parkar, and Jeremy Bracegirdle. Formal analysis: Rina Hannaford and Wayne Young. Investigation: Charlotte Hurst, Trent Olson, Jeremy Bracegirdle, and Nabil Parkar. Data curation: Nabil Parkar and Charlotte Hurst. Writing–original draft preparation: Charlotte Hurst, Gosia Zobel, Rina Hannaford, and Wayne Young. Writing–review and editing: Julie E. Dalziel, Rachel C. Anderson, and Jeremy Bracegirdle. Visualization: Rina Hannaford, Nabil Parkar, and Wayne Young. Supervision: Julie E. Dalziel and Rachel C. Anderson. Project administration: Charlotte Hurst. All authors have read and agreed to the final version of the manuscript.

## Ethics Statement

This study was approved by the AgResearch Animal Ethics Committee (Hamilton, New Zealand; AEC approval number 15245).

## Peer Review

The peer review history for this article is available at https://publons.com/publon/10.1002/brb3.70621

## Supporting information




**Supporting Material**: brb370621‐sup‐0001‐SuppMat.docx

## Data Availability

The data that support the findings of this study are available from the corresponding author upon reasonable request. We have deposited our microbiome data at NCBI bioproject ID: PRJNA1127265 ‐ SRA ‐ NCBI
