# Supplementary material for: Social Isolation Induces Sex‐Specific Differences in Behavior and Gut Microbiota Composition in Stress‐Sensitive Rats
Source: Brain Behav. 2025 Jun 10;15(6):e70621. doi: 10.1002/brb3.70621 (PMC12152274; doi:10.1002/brb3.70621)
Supplement: Supplementary file 1 — Supporting Material: brb370621‐sup‐0001‐SuppMat.docx [file BRB3-15-e70621-s001.docx]

**
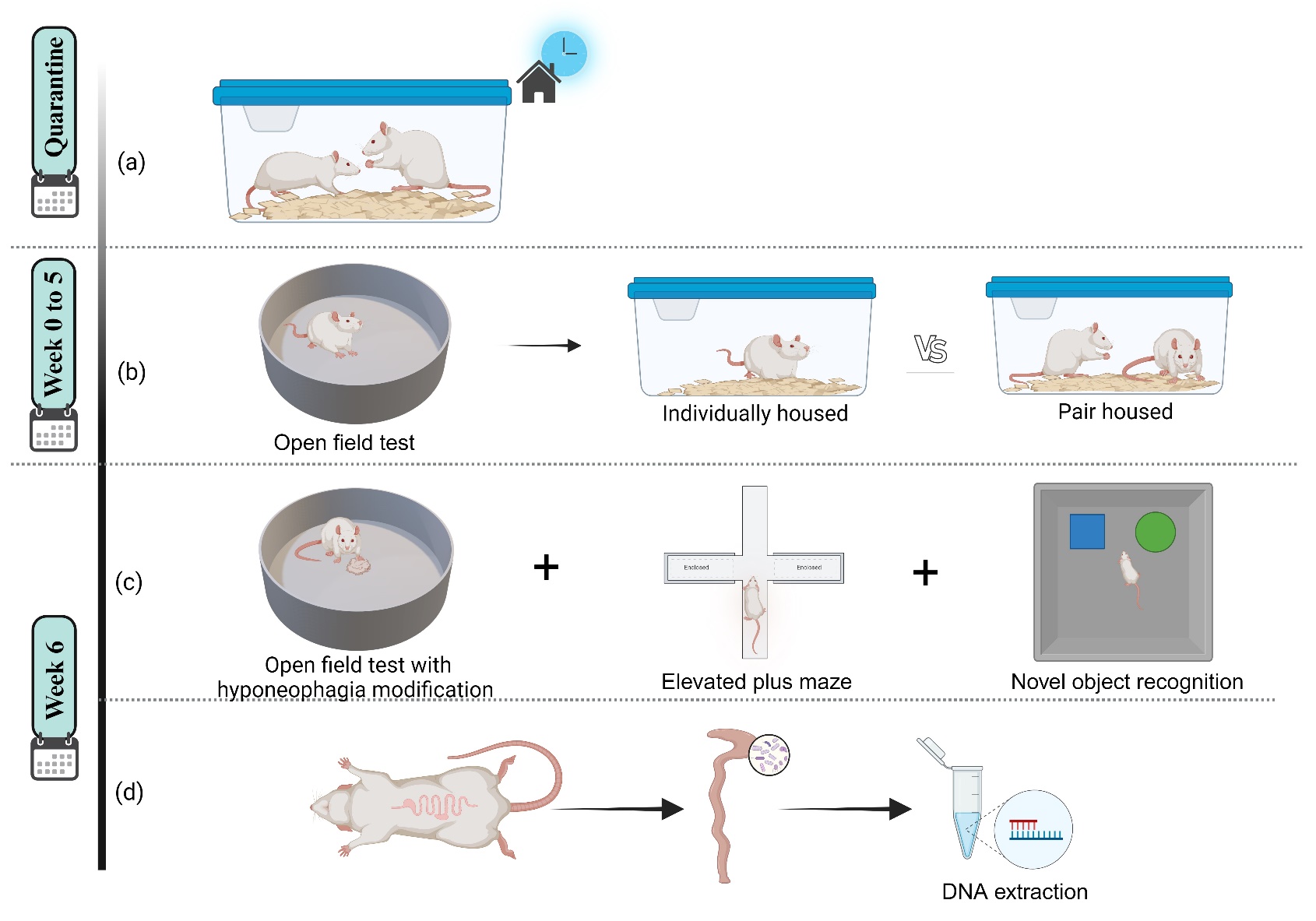
**

**Supplementary 1.** Study design and methods. Created with biorender.com

**Supplementary 2**. Behavioural data analysis

Baseline anxiety - The duration of time that each rat spent in the centre of the standard OFT arena (prior to enrolment in housing treatments) was used as an indicator of pre-trial baseline anxiety and included as a covariate.

OFTmod - A linear mixed model, fitted using Residual Maximum likelihood with the lmer function from the lme4 package (35), was used to model time spent in the centre of the OFTmod (where food located), with cage a random effect. The fixed effects were study block, sex, housing, baseline anxiety, and biologically relevant interactions.

**
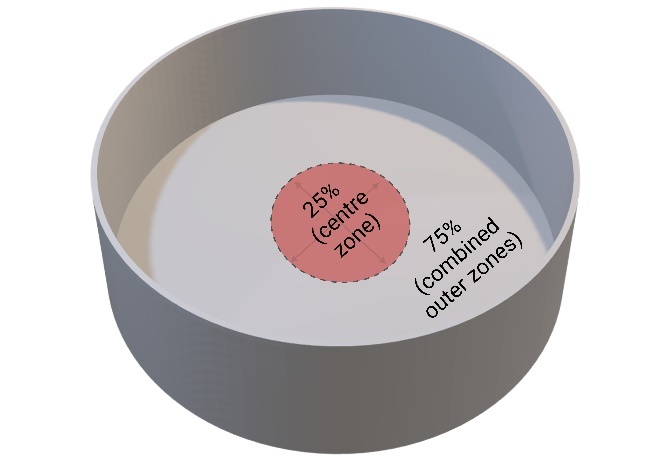
**

Image to demonstrate the combination of the outer zones of the OFTmod. Created with biorender.com

EPM - The effect of housing treatment on the proportion of time spent in each zone of the EPM (close/open arms, centre) was investigated using a generalized linear model, with a Beta distribution for the response variable and a logit link function with the glmmTMB function from the glmmTMB package (36). Other covariates included in the model were study block, sex, baseline anxiety, and biologically relevant interactions.

NOR - The preference of the left object over the right object on the familiarisation day (exploration of familiar arena with two identical objects) was used as a measure of each rat’s ‘sidedness’. On test day (exploration of familiar arena with one familiar and one novel object), the time spent with the novel object divided by the total exploration time for both objects was calculated (recognition index; (37)). A Beta regression mean model with a logit link function (36) was used to assess whether there was evidence that the recognition index was affected by the study block, whether the novel object was placed on the same side as the rat’s preferred side, sex, housing, baseline anxiety, and biologically relevant interactions.

(b)

(a)


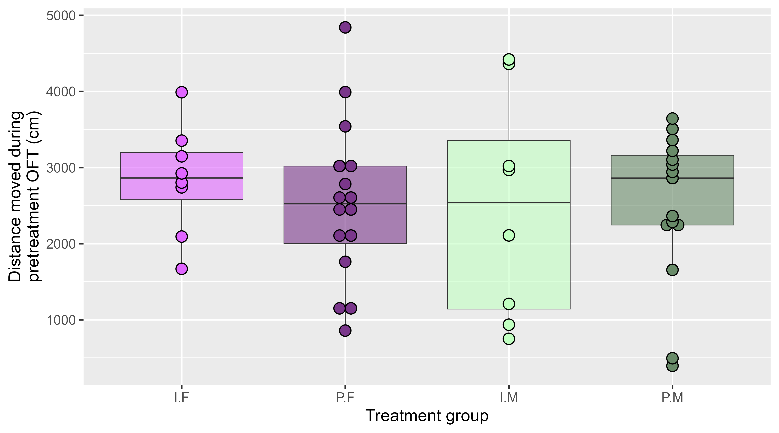

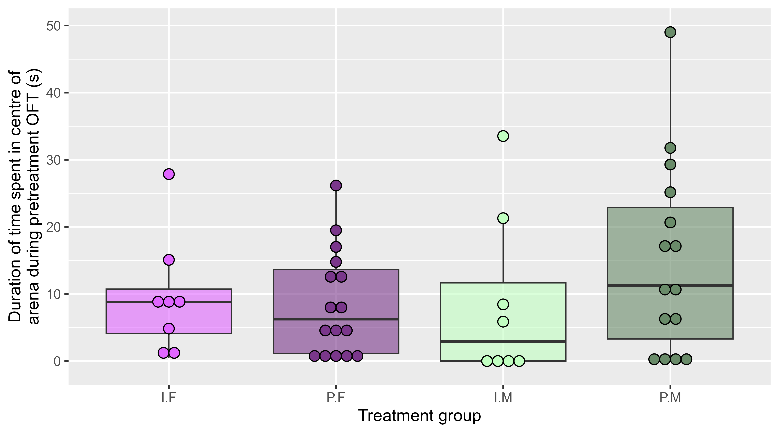


**Supplementary 3.** Initial Open Field Test

Duration of time spent in the centre (A) and total distance moved (B) in the pre-treatment open field test (OFT) arena for individually housed females (I.F, n = 8), pair-housed females (P.F, n = 16), individually housed males (I.M, n = 8) and pair-housed males (P.M, n = 16). Tests were 10 min in length. The ANOVA found no evidence of any differences in time spent in the centre due to either housing (p=0.625) or sex (0.778), and the no evidence of any differences in distance moved due to either housing (p= 0.6939) or sex (p= 0.6436).

**Supplementary Table 1**. KEGG (Kyoto Encyclopedia of Genes and Genomes) level 3 categories in the caecum with significant differences (ANCOM-BC, q<0.05) in relative abundance between female rats housed individually (IF) or in pairs (PF). Mean percent ± standard error of means.

| KEGG function/category | IF | PF | *p* value | *q* value | W^1^ |
| --- | --- | --- | --- | --- | --- |
| Xenobiotics biodegradation and metabolism; ko00362 Benzoate degradation | 0.07 ± 0.001 | 0.08 ± 0.002 | <0.001 | <0.001 | 5.875 |
| Brite Hierarchies; Protein families: signaling and cellular processes; ko02048 Prokaryotic defense system | 1.95 ± 0.018 | 2.11 ± 0.031 | <0.001 | <0.001 | 4.835 |
| Brite Hierarchies; Protein families: signaling and cellular processes; ko02035 Bacterial motility proteins | 0.05 ± 0.002 | 0.06 ± 0.003 | <0.001 | <0.001 | 4.584 |
| Environmental Information Processing; Signal transduction; ko02020 Two-component system | 3.22 ± 0.044 | 3.60 ± 0.069 | <0.001 | <0.001 | 4.564 |
| Brite Hierarchies; Protein families: signaling and cellular processes; ko02044 Secretion system | 0.57 ± 0.01 | 0.64 ± 0.011 | <0.001 | <0.001 | 4.47 |
| Not Included in Pathway or Brite; Unclassified: signaling and cellular processes; ko99978 Cell growth | 2.11 ± 0.039 | 2.40 ± 0.049 | <0.001 | <0.001 | 4.464 |
| Environmental Information Processing; Membrane transport; ko03070 Bacterial secretion system | 0.33± 0.003 | 0.36 ± 0.007 | <0.001 | <0.001 | 4.411 |
| Brite Hierarchies; Protein families: genetic information processing; ko03000 Transcription factors | 2.33 ± 0.018 | 2.48 ± 0.033 | <0.001 | 0.002 | 3.882 |
| Brite Hierarchies; Protein families: signaling and cellular processes; ko01504 Antimicrobial resistance genes | 0.24 ± 0.004 | 0.25 ± 0.004 | <0.001 | 0.002 | 3.786 |
| Brite Hierarchies; Protein families: genetic information processing; ko03036 Chromosome and associated proteins | 1.51 ± 0.015 | 1.58 ± 0.018 | <0.001 | 0.002 | 3.779 |
| Amino acid metabolism; ko00220 Arginine biosynthesis | 0.32 ± 0.004 | 0.33 ± 0.004 | <0.001 | 0.002 | 3.744 |
| Carbohydrate metabolism; ko00650 Butanoate metabolism | 0.45 ± 0.008 | 0.51 ± 0.014 | <0.001 | 0.002 | 3.74 |
| Genetic Information Processing; ko04141 Protein processing in endoplasmic reticulum | 0.16 ± 0.003 | 0.17 ± 0.002 | <0.001 | 0.003 | 3.652 |
| Environmental Information Processing; Signal transduction; ko04075 Plant hormone signal transduction | 0.003 ± 0.001 | 0.004 ± 0.001 | <0.001 | 0.005 | 3.49 |
| Carbohydrate metabolism; ko00030 Pentose phosphate pathway | 0.79 ± 0.012 | 0.82 ± 0.009 | 0.002 | 0.013 | 3.142 |
| Metabolism; Energy metabolism; ko00680 Methane metabolism | 0.30 ± 0.004 | 0.32 ± 0.005 | 0.002 | 0.015 | 3.096 |
| Environmental Information Processing; Membrane transport; ko02010 ABC transporters | 4.91 ± 0.07 | 5.13 ± 0.072 | 0.002 | 0.015 | 3.082 |
| Amino acid metabolism; ko00360 Phenylalanine metabolism | 0.19 ± 0.007 | 0.21 ± 0.005 | 0.002 | 0.015 | 3.042 |
| Genetic Information Processing; Replication and repair; ko03430 Mismatch repair | 0.55 ± 0.007 | 0.57 ± 0.009 | 0.003 | 0.016 | 3.018 |
| Not Included in Pathway or Brite; Unclassified: metabolism; ko99981 Carbohydrate metabolism | 0.08 ± 0.003 | 0.09 ± 0.002 | 0.005 | 0.025 | 2.823 |
| Brite Hierarchies; Protein families: signaling and cellular processes; ko04812 Cytoskeleton proteins | 0.10 ± 0.003 | 0.11 ± 0.003 | 0.006 | 0.032 | 2.728 |
| Not Included in Pathway or Brite; Unclassified: genetic information processing; ko99974 Translation | 0.06 ± 0.002 | 0.07 ± 0.002 | 0.008 | 0.04 | 2.636 |
| Amino acid metabolism; ko00270 Cysteine and methionine metabolism | 1.25 ± 0.014 | 1.27 ± 0.012 | 0.009 | 0.041 | 2.625 |
| Carbohydrate metabolism; ko00051 Fructose and mannose metabolism | 0.73 ± 0.012 | 0.75 ± 0.012 | 0.009 | 0.042 | 2.608 |
| Metabolism of other amino acids; ko00440 Phosphonate and phosphinate metabolism | 0.09 ± 0.002 | 0.09 ± 0.001 | 0.009 | 0.042 | 2.599 |
| Lipid metabolism; ko00561 Glycerolipid metabolism | 0.43 ± 0.006 | 0.44 ± 0.004 | 0.011 | 0.047 | 2.536 |
| Not Included in Pathway or Brite; Unclassified: genetic information processing; ko99973 Transcription | 0.13 ± 0.002 | 0.14 ± 0.002 | 0.012 | 0.048 | 2.517 |
| Not Included in Pathway or Brite; Poorly characterized; ko99997 Function unknown | 3.70 ± 0.036 | 3.77 ± 0.026 | 0.012 | 0.048 | 2.512 |
| Brite Hierarchies; Protein families: signaling and cellular processes; ko04147 Exosome | 0.11 ± 0.002 | 0.11 ± 0.003 | 0.012 | 0.048 | 2.508 |
| Brite Hierarchies; Protein families: metabolism; ko01005 Lipopolysaccharide biosynthesis proteins | 0.17 ± 0.004 | 0.18 ± 0.005 | 0.013 | 0.049 | 2.475 |
| Not Included in Pathway or Brite; Unclassified: signaling and cellular processes; ko99992 Structural proteins | 0.27 ± 0.008 | 0.24 ± 0.007 | 0.014 | 0.049 | -2.468 |
| Metabolism of cofactors and vitamins; ko00750 Vitamin B6 metabolism | 0.12 ± 0.003 | 0.11 ± 0.002 | 0.013 | 0.049 | -2.471 |
| Genetic Information Processing; ko03018 RNA degradation | 0.99 ± 0.011 | 0.92 ± 0.015 | 0.013 | 0.049 | -2.472 |
| Brite Hierarchies; Protein families: signaling and cellular processes; ko02042 Bacterial toxins | 0.16 ± 0.004 | 0.15 ± 0.003 | 0.011 | 0.047 | -2.542 |
| Brite Hierarchies; Protein families: signaling and cellular processes; ko03200 Viral proteins | 0.01 ± 0.002 | 0.009 ± 0.001 | 0.008 | 0.04 | -2.636 |
| Glycan biosynthesis and metabolism; ko00515 Mannose type O-glycan biosynthesis | 0.03 ± 0.002 | 0.02 ± 0.001 | 0.004 | 0.022 | -2.871 |
| Brite Hierarchies; Protein families: genetic information processing; ko04121 Ubiquitin system | 0.004 ± 0.001 | 0.003 ± 0.001 | 0.004 | 0.021 | -2.893 |
| Brite Hierarchies; Protein families: metabolism; ko01002 Peptidases and inhibitors | 1.99 ± 0.029 | 1.86 ± 0.025 | 0.003 | 0.017 | -2.963 |
| Carbohydrate metabolism; ko00630 Glyoxylate and dicarboxylate metabolism | 0.71 ± 0.005 | 0.66 ± 0.012 | 0.003 | 0.017 | -2.986 |
| Metabolism of cofactors and vitamins; ko00130 Ubiquinone and other terpenoid-quinone biosynthesis | 0.14 ± 0.008 | 0.11 ± 0.006 | 0.003 | 0.016 | -3.002 |
| Biosynthesis of other secondary metabolites; ko00998 Biosynthesis of various secondary metabolites - part2 | 0.003 ± 0.001 | 0.002 ± 0.001 | 0.002 | 0.015 | -3.064 |
| Lipid metabolism; ko00121 Secondary bile acid biosynthesis | 0.012 ± 0.001 | 0.009 ± 0.001 | 0.002 | 0.013 | -3.139 |
| Metabolism of cofactors and vitamins; ko00830 Retinol metabolism | 0.006 ± 0.001 | 0.004 ± 0.001 | 0.002 | 0.013 | -3.14 |
| Genetic Information Processing; ko04120 Ubiquitin mediated proteolysis | 0.009 ± 0.001 | 0.007 ± 0.001 | 0.001 | 0.01 | -3.246 |
| Biosynthesis of other secondary metabolites; ko00940 Phenylpropanoid biosynthesis | 0.01 ± 0.001 | 0.007 ± 0.001 | 0.001 | 0.008 | -3.327 |
| Lipid metabolism; ko00140 Steroid hormone biosynthesis | 0.007 ± 0.001 | 0.005 ± 0.001 | <0.001 | 0.004 | -3.541 |
| Not Included in Pathway or Brite; Unclassified: metabolism; ko99999 Others | 0.011 ± 0.001 | 0.007 ± 0.001 | <0.001 | 0.002 | -3.745 |
| Glycan biosynthesis and metabolism; ko00510 N-Glycan biosynthesis | 0.034 ± 0.001 | 0.028 ± 0.001 | <0.001 | 0.002 | -3.789 |
| Brite Hierarchies; Protein families: metabolism; ko01011 Peptidoglycan biosynthesis and degradation proteins | 0.07 ± 0.003 | 0.054 ± 0.003 | <0.001 | <0.001 | -4.321 |

^1^ Test statistic of Analysis of Compositions of Microbiomes with Bias Correction (ANCOM-BC) package

**Supplementary Table 2.** Comparison of prior Elevated Plus Maze tests completed at the Ulyatt Reid Facility, AgResearch, Palmerston North New Zealand. Time spent in test arena was 5 minutes for each trial. Housing type: IH – Individually housed, PH – Pair-housed.

**[1]** Strain comparison study 2019 – Unpublished data

**[2]** ^1^

**[3]** Current study

| **Trial** | **[1]** | **[1]** | **[2]** | **[3]** |
| --- | --- | --- | --- | --- |
| **Strain** | SD | WKY | WKY | WKY |
| **Sex** | Male | Male | Male | Male & Female |
| **Housing type** | IH | IH | IH | IH and PH |
| **Diet** | Soy based | Soy based | Casein based | Soy based |
| **Age (wks)** | 10-12 | 10-12 | 23-24 | 8-10 |
| Total distance moved (cm) | 1700 | 900 |  | 800 |
| Mean velocity (cm/s) | 5.75 | 3 | 2 | 3 |
| Open arms (cumulative duration) (s) | 50 | 30 | < 5 | 26 |
| Closed arms (cumulative duration) (s) | 205 | 180 | 260 | 193 |

^1^ Dalziel, J. E. *et al.* A Diet Enriched with Lacticaseibacillus rhamnosus HN001 and Milk Fat Globule Membrane Alters the Gut Microbiota and Decreases Amygdala GABA a Receptor Expression in Stress-Sensitive Rats. *International Journal of Molecular Sciences* **24**, 10433 (2023). <https://doi.org/https://doi.org/10.3390/ijms241310433>

**Supplementary Table 3.** Comparison of prior Open field tests completed at the Ulyatt Reid Facility, AgResearch, Palmerston North New Zealand. Time spent in test arena was 10 minutes for each trial. Housing type: IH – Individually housed, PH – Pair-housed.

[1] Strain comparison study 2019 – Unpublished data

[2] ^1^

[3] Current study – Outer zones (50%, 75% & 100%) combined for analysis

[4] ^2^

| **Trial** | **[1]** | **[1]** | **[2]** | **[3]** | **[4]** | **[4]** |
| --- | --- | --- | --- | --- | --- | --- |
| **Strain** | SD | WKY | WKY | WKY | SD | WKY |
| **Sex** | Male | Male | Male | Male & Female | Male | Male |
| **Housing type** | IH | IH | IH | IH and PH | IH | IH |
| **Diet** | Soy based | Soy based | Casein based | Soy based | Casein based | Casein based |
| **Age (wks)** | 10-12 | 10-12 | 23-24 | 8-10 | 10 | 10 |
| Time in zones (%) |  | | | | | |
| 25% zone | 3 | 2 | 1 | 3 | 4 | 1 |
| 50% zone | 4 | 2 | 1 | 97 | 7 | 2 |
| 75% zone | 21 | 10 | 1 |  | 36 | 12 |
| 100% zone | 70 | 86 | 97 |  | 52 | 85 |
| Total distance moved (cm) | 2034 | 1900 | 600 | 2500 | 5200 | 1500 |
| Mean velocity (cm/s) | 3.4 | 1.55 | 1 | 4.1 | 8.8 | 2.5 |

^1^ Dalziel, J. E. *et al.* A Diet Enriched with Lacticaseibacillus rhamnosus HN001 and Milk Fat Globule Membrane Alters the Gut Microbiota and Decreases Amygdala GABA a Receptor Expression in Stress-Sensitive Rats. *International Journal of Molecular Sciences* **24**, 10433 (2023). <https://doi.org/https://doi.org/10.3390/ijms241310433>

^2^ Bassett, S. A. *et al.* Metabolome and microbiome profiling of a stress-sensitive rat model of gut-brain axis dysfunction. *Scientific Reports* **9**, 14026 (2019). <https://doi.org/https://doi.org/10.1038/s41598-019-50593-3>
